# Microfluidic Devices in Advanced *Caenorhabditis elegans* Research

**DOI:** 10.3390/molecules21081006

**Published:** 2016-08-02

**Authors:** Muniesh Muthaiyan Shanmugam, Tuhin Subhra Santra

**Affiliations:** 1 Institute of Molecular and Cellular Biology, Department of Life Science, National Tsing Hua University, Hsinchu 30013, Taiwan; 2 Department of Engineering Design, Indian Institute of Technology Madras, Chennai 600 036, India; santra.tuhin@gmail.com

**Keywords:** worm chips, microfluidics devices, *C. elegans*, worm immobilization, model organism, nematode worm

## Abstract

The study of model organisms is very important in view of their potential for application to human therapeutic uses. One such model organism is the nematode worm, *Caenorhabditis elegans*. As a nematode, *C. elegans* have ~65% similarity with human disease genes and, therefore, studies on *C. elegans* can be translated to human, as well as, *C. elegans* can be used in the study of different types of parasitic worms that infect other living organisms. In the past decade, many efforts have been undertaken to establish interdisciplinary research collaborations between biologists, physicists and engineers in order to develop microfluidic devices to study the biology of *C. elegans*. Microfluidic devices with the power to manipulate and detect bio-samples, regents or biomolecules in micro-scale environments can well fulfill the requirement to handle worms under proper laboratory conditions, thereby significantly increasing research productivity and knowledge. The recent development of different kinds of microfluidic devices with ultra-high throughput platforms has enabled researchers to carry out worm population studies. Microfluidic devices primarily comprises of chambers, channels and valves, wherein worms can be cultured, immobilized, imaged, etc. Microfluidic devices have been adapted to study various worm behaviors, including that deepen our understanding of neuromuscular connectivity and functions. This review will provide a clear account of the vital involvement of microfluidic devices in worm biology.

## 1. Introduction

Microfluidic/nanofluidic systems generally employ the principles of fluidics at the micro- or nanoscale level. Microfluidic devices provide greater control over microenvironments, with simple mechanical, electrical, chemical or computer-controlled units that enhance preferences for microfluidic devices in various kinds of research [1]. Researchers can use these devices to improve model organism handling and therefore employ them in a diverse spectrum of studies [2]. Microfluidics research has focused on developing devices and tools for facilitating research fields such as biology, medicine, etc. Although one can use the microfluidic approach to study diverse sets of model organisms in biological research (including cell lines [3,4,5,6,7,8], *Drosophila* spp. [9], fish [10], etc. [11,12,13,14]) this review will concentrate only on worm-based chips, particularly those involving *Caenorhabditis elegans* (*C. elegans*).

*C. elegans* is a versatile, soil-dwelling nematode, which is transparent and very small in size (~1.5 mm in length) with a short life cycle (approximately three days for development from zygote to young adult). The adult worm can survive up to 30 days (depending on the incubation temperature) and can produce some 300 to 350 progeny [15]. Sydney Brenner, who was awarded the Nobel Prize in Physiology or Medicine in 2002, introduced this powerful model organism in the late 1960s. *C. elegans* feed on soil bacteria in their natural habitat, while in the laboratory environment they are fed special strains of *E. coli*. The complete cell lineage of the development and whole genome sequence of *C. elegans* is available, which further enhances the popularity of this worm as a versatile research model organism. The worm’s transparent body enables in vivo visualization of fluorescently tagged protein expression and other in vivo analysis [16]. Further, the genetics of the worm is completely explored, rendering *C. elegans* a powerful tool for forward genetic screening/analysis and other genetic studies [17]. *C. elegans* shares great homology with the human genome while remaining a very primitive model organism with several conserved pathways, for example an insulin signaling pathway, a simple nervous system etc. thus properly balancing the simplicity and complexity of the organism during evolution. Researchers also use this worm to understand host-pathogen interactions [18,19] and to screen drugs for several related parasitic worms that infect mammalian livestock and human beings [20].

Over the past decade, the use of microfluidic worm chips has accelerated several aspects of worm research. There are number of advantages in using microfluidic platforms for worm research, such as easy handling and manipulation of worms at the cellular level, quick immobilization of the worms for live imaging with less damages to the worm and without the use of harmful chemical agents, rapid screening of several hundreds or thousands of worms for the recovery of a particular worm with the desired phenotype, sorting of the mixed worm population into different groups with particular characteristics, precise microsurgery on microscopic organisms at the micro- or nanoscale level, and controlled manipulation of worms’ living microenvironment (such as the available amount of food, drug, etc.) so as to study various short-term and long-term cellular and behavioral responses. As a result of abovementioned advantages researchers are highly motivated to develop new designs of microfluidic systems for high performance.

Apart from the introduction of new designs (in order to make the assays high-throughput) in microfluidic devices to study a particular phenomenon in worm, new concepts for analyzing worms using worm chips are also being published, for example, the introduction of the concept of ‘worm treadmill’ by Chuang et al. [21], where the worms are subjected to controlled exercise using electrotaxis behavior to study the effect of exercise on neurodegeneration [21]. As an inter-disciplinary field, which combines biology and microfluidics, several reviews are constantly being published to explain the use of this elegant collaboration to the broader scientific communities. The emphasis of each article differs greatly, and while some focus on introducing microfluidics and worm biology to scientists in other research disciplines, like chemistry [22], some focus on the involvement of microfluidic devices in whole-organisms screening [12], some reviews focus on explaining the involvement of microfluidic chips in facilitating handling of differential organisms and tissue samples [11,12,13,23], whereas some reviews focus only on a particular organism such as application of microfluidic devices in *C. elegans* [24,25]. This review will emphasize a clear understanding of microfluidic systems in worm research and its potential applications. Further, it will also elaborate some important worm chips along with recently-developed worm chips and their future research directions.

## 2. Basic Microfluidic Device Construction in *C. elegans* Research

The identification of a soft silicone elastomer, polydimethylsiloxane (PDMS), as a flexible, stretchable, nontoxic, inert, biocompatible, gas-permeable and optically transparent material has opened up a new direction for the fabrication of microfluidic or bio-micro electromechanical systems (Bio-MEMS) devices, which researchers can use in a variety of biological investigations. Microchips or microfluidic devices are designed and fabricated in such a way that they can be used for culturing and studying various biological, living entities, such as cells and model organisms [1,2,7,25], in microfluidic environments. Successful development of microfluidic devices involves three distinct stages: (1) designing the device; (2) fabricating the device and; finally, (3) testing the efficiency of the device. Microfluidic chips come in a variety of designs and their design requirements depend on the biological investigation objectives of the particular study. Researchers can design a particular microfluidic system by employing any of the vast amount of available 3D modeling software packages (for example, AutoCAD^®^ (for computer-aided design), Shape3D, Adobe^®^, etc.). Further, finely crafted PDMS surfaces are engineered to mimic model organisms’ native habitats, thereby improving research efficacy [26]. Similarly, the choice of fabrication approach, such as soft lithography, multilayer lithography, silicon bulk and surface micromachining, polymer machining, etc., depend on user requirements [2]. Besides existing methods and material combinations, researchers are developing new methods to make more complex structures possible [27,28]. The fabrication of worm chips is cost-effective and less time-consuming than other approaches. Finally, the testing phase involves the testing of the microfluidic device’s efficiency, addressing the various questions of individual research fields. One can find more detailed information about the chip fabrication and processing steps in the corresponding literature [25,29,30,31,32,33].

## 3. Classification of Microfluidic Devices Used for *C. elegans* Research

In order to overcome several technical difficulties in handling *C. elegans* and to enhance various aspects of scientific understanding, apart from non-microfluidic systems, researchers have developed numerous microfluidic devices and continue to develop more. However, it is not possible to clearly categorize these devices into particular groups, as these devices have multifunctional capabilities and interchangeable applications. Experts have differential preferences for classification of these devices based on utility, biological and physical characteristics—for example, Chronis divided worm chips into two categories, namely single worm chips and worm population chips [2]. Nevertheless, in order to provide a basic understanding of the various microfluidic devices, we present below a simple classification of these devices based on their applications in worm research. Microfluidic devices are used for the following applications: (a) immobilization and imaging; (b) metabolic research studies; (c) behavioral assays; (d) drug screening and toxicology studies; (e) microsurgery; and (f) worm/*C. elegans* sorting.

## 4. Microfluidic Devices for Worm Applications

Extensive efforts have been applied in the past few decades to develop numerous worm chips for various research applications. These worm chips differ greatly in their application, structural design and complexity [1,2,13,25]. The following are some of the important and recent microfluidic worm chips used for research and development.

### 4.1. Immobilization and Imaging

*C. elegans* are transparent animals, which allows any researcher to perform in vivo visualization of a fluorescently tagged protein that is transgenically expressed and serves as a greater, yet simple, in vivo model organism [16]. Such live observations facilitate detailed understanding of several subcellular functions and molecular interactions of the fluorescently tagged protein. In order to achieve successful imaging of living worms, it is absolutely necessary to immobilize the worms. A live worm swims constantly in the buffer solution in which it is suspended. However, the need of immobilizing the worm also depends on experimental needs—for example, neuronal activity imaging in certain cases requires a non-immobilized worm, like calcium imaging so as to understand neuronal activity and behavior [34]. Methods and software have been developed to facilitate the imaging of mobile worms [34]. A simple, cost-effective method of immobilizing a worm for a short time, so as to achieve in vivo imaging, is followed by all *C. elegans*-based research laboratories. According to this method, researchers typically sandwich the worms between a 2% agarose-coated glass slide and a cover-glass, along with an appropriate buffer or M9 buffer (3 g KH_2_PO_4_, 6 g Na_2_HPO_4_, 5 g NaCl, 1 mL 1 M MgSO_4_, H_2_O to 1 L, sterilize by autoclaving) containing an anesthetic, such as levamisole, tetramisole, etc. [35]. Unfortunately, using this method for live in vivo imaging is not possible over a longer period, as the worms’ vital signs deteriorate and the anesthesia is shown to affect the cellular physiology [20]. Further, researchers cannot use this method for screening purposes, as it is almost impossible to recover a particular worm from the sandwich. In order to overcome these disadvantages of the above mentioned procedure, several types of methods have been developed, whereby researchers can perform live worm imaging for longer time periods without anesthesia, or screen the worm for a specific phenotype and recover it for further analysis. However, it was difficult to overcome all the disadvantages simultaneously until microfluidic devices for long-term immobilization and imaging were invented and developed. All new procedures following the use of microfluidic devices reduce the damage caused to the worms, though not completely. Further new microfluidic devices always aim at increasing performance and thereby providing quicker recovery and survival rates for the worms.

**Figure 1:**
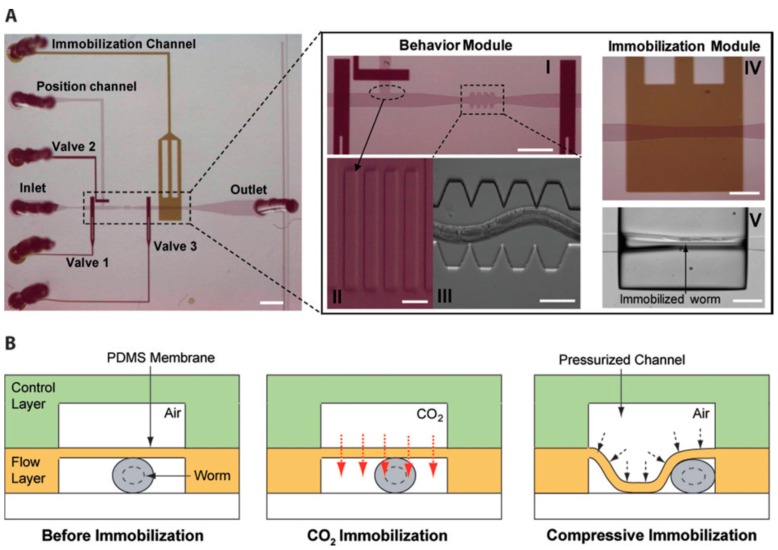
(**A**) Shows the microfluidic device developed by Chokshi et al. [36]. I, II and III show magnified views of the behavior modules, via which one can observe and analyze the worm’s motility pattern, while IV and V show the immobilization modules, where one can immobilize and image the worm; (**B**) Pictorial representation of the two methods of worm immobilization. Reprinted with permission from the Royal Society of Chemistry.

Chokshi et al [36]. developed a simple worm chip system to efficiently immobilize the worms in addition to studying the motility behavior of *C. elegans*. This system involves two layers of PDMS on a glass slide with the appropriate chambers and channels for fluid flow (Figure 1). The worm is immobilized via two different techniques: the first utilizes the diffusion property of PDMS to diffuse CO_2_ from the upper PDMS channel to the lower channel containing the worm, thereby immobilizing the worm; the second employs the collapsible nature of PDMS to mechanically compress the worm by increasing the air pressure on the upper PDMS fluidic channel [36]. Following immobilization of the worm of up to an hour, Chokshi et al. [36] quantified the damage caused by the immobilization by evaluating the locomotive speed of the worm. They found that immobilization using CO_2_ caused less damage to the worm than the application of mechanical pressure. The average speed of the worm after immobilization for an hour was reduced to 0% and 70%, respectively, upon remobilization using the collapsible PDMS and CO_2_ methods. Mechanical immobilization of worms using the collapsible property of PDMS and then successful imaging of intracellular transport was performed, demonstrating the easy handling and versatility of microfluidic worm chips [9,37].

**Figure 2:**
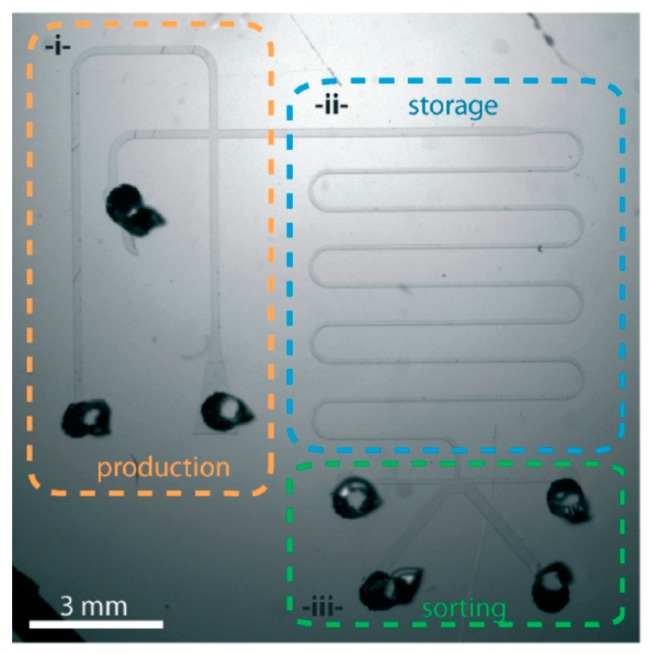
The worm chip developed by Aubry et al. [38], with its different modules. Reprinted with permission from the Royal Society of Chemistry.

**Figure 3:**
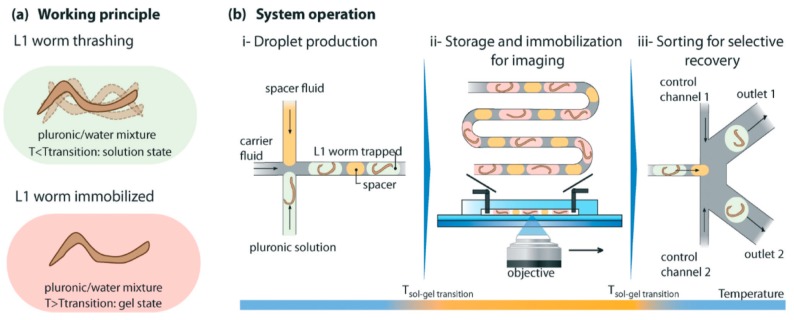
A schematic representation of the method of immobilization, imaging and sorting of worms developed by Aubry et al. [38]. (**a**) The working principle whereby the worm can thrash in a Pluronic liquid medium and is immobilized in the gel medium; (**b**) the operating principle whereby Aubry et al. separated a single worm at (i), store, immobilize and image the worm (ii) and sort the worm (iii). This illustration also indicates the corresponding change in temperature in different modules. Reprinted with permission from the Royal Society of Chemistry.

Although microfluidic systems using valves, channels and chambers are very efficient for the handling of adult worms, studies involving the larval stage of worms are challenging, given that they are much smaller than adult worms. Very small first larval stage (L1) worms can easily clog the microfluidic channels and have less efficient trapping. In order to overcome the challenge of larval stage worms, Aubry et al. combined the temperature-sensitive reversible gelation of Pluronic F27 with microfluidic channels [38]. Initially, this chip works by trapping a single worm in the production unit with Pluronic F27 (Figure 2). As the worm enters the storage unit of the chip, one can adjust the temperature to solidify the gel, resulting in immobilization of the worm and thereby allowing for efficient imaging. Finally, researchers can sort the worm in the sorting unit of the chip. We present a schematic representation of a working model of this device in Figure 3.

Cornaglia et al. introduced a device for immobilization and imaging of *C. elegans* embryos to study their development and the molecular changes that take place during embryogenesis. This device consists of a worm culture chamber, where one can culture adult worms, and an array of micro-compartments called embryo-chambers, where researchers immobilize a single embryo and examine it in real time throughout its development. An array of embryo-chambers in this device also allows for the performance of high throughput (HTP) analysis by permitting researchers to analyze several embryos at the same time [39].

**Figure 4:**
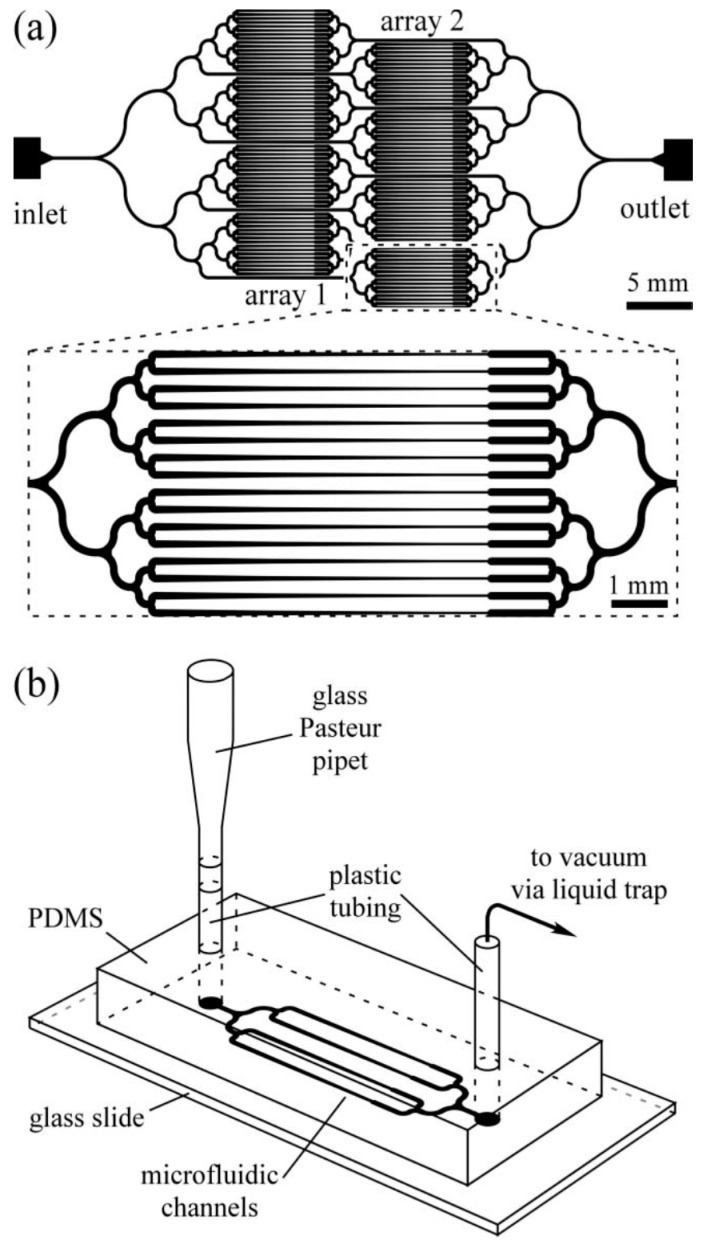
(**a**) The design developed by Hulme et al. [40] for an array of worm immobilization units for HTP immobilization, which researchers can use for synchronized imaging or axotomy; (**b**) a pictorial representation of the microfluidic system developed by Hulme et al., indicating different parts of the device. Reprinted with permission from the Royal Society of Chemistry.

**Figure 5:**
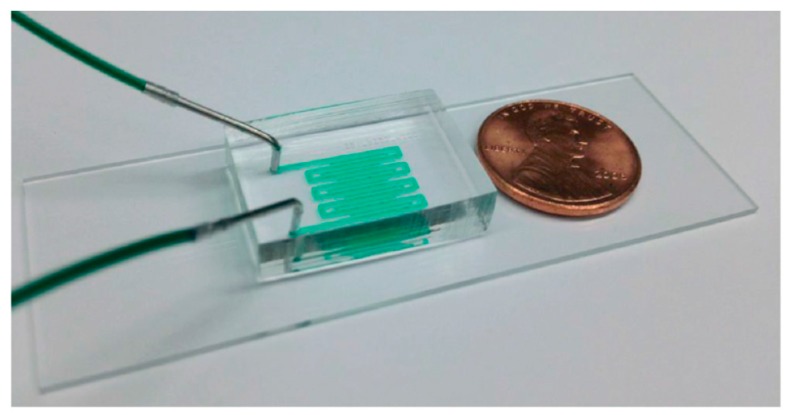
The microfluidic device developed by Lee et al. [41] for the immobilization of an array of worms for HTP analysis. Reprinted with permission from the Royal Society of Chemistry.

**Figure 6:**
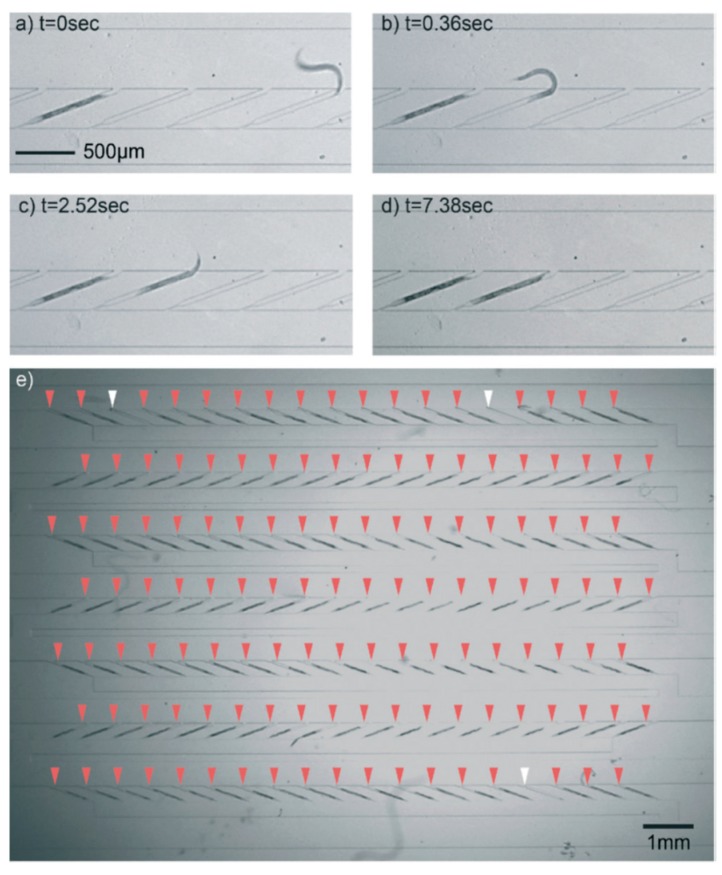
A magnified view of an array of microfluidic channels and immobilized worms from the device shown in Figure 5. (**a**–**d**) The crawling and subsequent immobilization of worm in the microchannels at successive time points; (**e**) an array of immobilized worms in microchannels, indicated by red arrowheads, and empty channels, indicated by white arrowheads. Reprinted with permission from the Royal Society of Chemistry.

Separate to the above-mentioned microfluidic devices, researchers have attempted to design HTP microfluidic platforms for the immobilization and imaging of several worms simultaneously, leading to some successful device development. For example, the microfluidic devices described by Hulme et al. [40] (Figure 4) and Lee et al. [41] (Figure 5 and Figure 6), use several arrays of narrow microchannels to entrap *C. elegans*, resulting in immobilization. Hulme et al. also monitored the survival of the worm after immobilization in their device, as they expected that the pressure exerted on the worm could possibly damage its cuticle and internal structures. By quantifying the number of days of survival of the worm and the number of offspring it created, Hulme et al. demonstrated that the worms exhibited normal function following immobilization. However, analysis at the gene expression level for stress response could provide a better understanding of the damage potentially caused by microfluidic devices to worms.

### 4.2. Metabolic Studies

**Figure 7:**
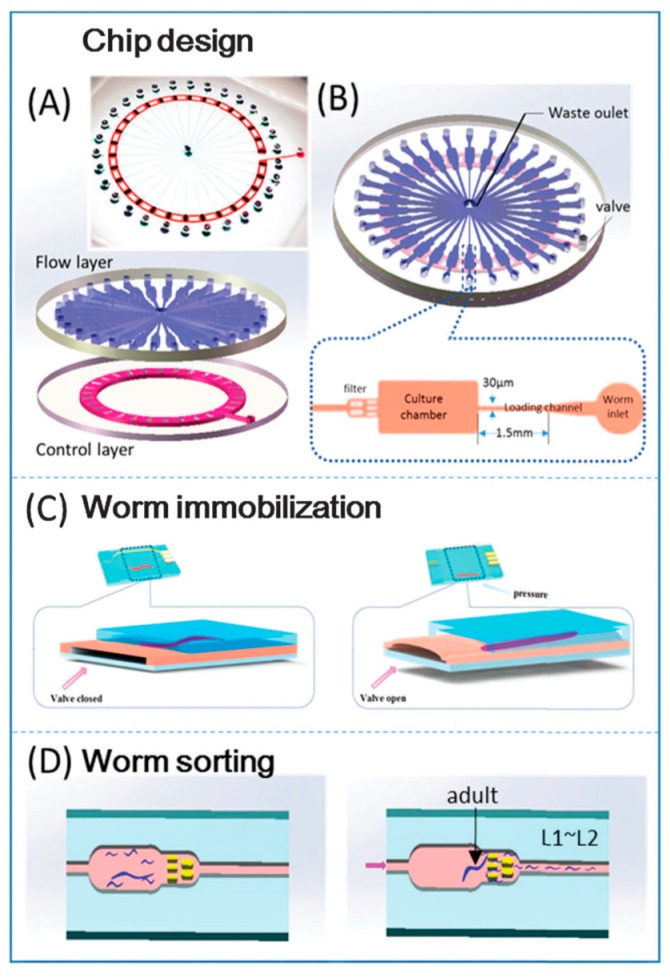
(**A**,**B**) The chip design developed by Zhu et al. [43] and the various layers and structures in the chip; (**C**) the methodology of worm immobilization via deformation of the bottom, control layer; (**D**) the methodology for separating young worms from the adult worm under observation. Reprinted with permission from the Royal Society of Chemistry.

A wide range of researchers use *C. elegans* as a model for studying and understanding metabolic diseases because the worm displays a sufficient homology to the human genome [42]. The addition of microfluidic systems can enhance metabolomics studies and is more advantageous for the development of treatment strategies for human diseases. Zhu et al. developed a multifunctional microfluidic chip which can perform multiparametric analysis and real-time metabolic monitoring for hyperglycemia throughout the life span of the worms [43]. The radial microfluidic system (Figure 7) is composed of transparent PDMS material and contains two layers. The upper layer is a fluidic chamber called the flow layer and the bottom layer is called the control layer, which researchers can use to immobilize the worms for imaging purposes. One can load the microchannel networks spreading from the center with worms, worm food and other active ingredients for worm treatment. Further, one can sort the newly hatched young worms through micro-pillar constructions, trapping adult worms for later analysis. Researchers can immobilize a worm for microscopic analysis by deforming the control layer membrane, thereby providing a reversible mechanical restriction. One can also use this chip for evaluation of the efficacy of anti-diabetic drugs. There is a very high chance that, in future, we will be able to develop more advanced microfluidic chips that would render *C. elegans* a good model for various metabolic disease studies.

### 4.3. Behavior Analysis

Researchers have developed numerous traditional behavioral assays for *C. elegans* since the introduction of *C. elegans* as a model organism. *C. elegans* have 302 neurons and the behavior of *C. elegans* in response to a particular stimuli reflects its neuronal and muscular functions and health [44]. Some common behaviors these researchers analyze include mechanosensation, osmotic avoidance, chemotaxis, electrotaxis, feeding response, thermal response, egg-laying, mating and reproductive behavior, learning and memory, defecation, etc. [44]. Following the development of microfluidics chips, we can obtain certain levels of accuracy in several of the behavioral assays—for example, dissection of precise concentrations of chemoattractant. Recent technological developments have provided advanced microfluidic chips that can help elucidate worm behaviors such as electrotaxis [45,46], chemotaxis [47,48], stress response behavior during crowding [49,50], neuronal pathway and subsequent behavioral responses [51], host invasion behavior [19], etc.

Although examination of chemotaxis behavior is a very common traditional assay (for example, quadrant assay [52]), the calculation of the accurate concentration or development of proper concentration gradient of chemical stimuli employed is, in most cases, difficult to determine. Wang et al. developed a microfluidic/nanofluidic device in which the nanochannels can develop a concentration gradient to test the chemical [47].

**Figure 8:**
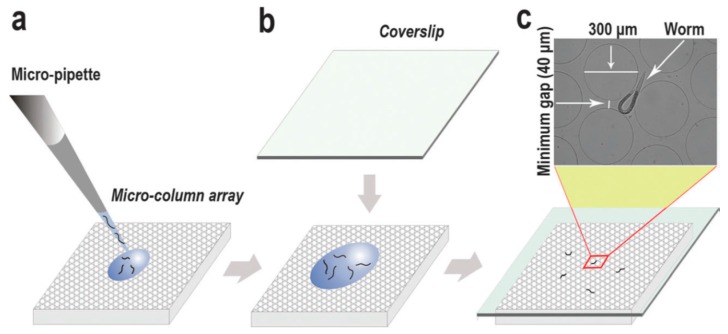
An array of micro-columns-based worm chip developed by Wang et al. [50] and the flow chart of the experimental procedure. (**a**) A pictorial representation of a worm chip in the process of worm-loading; (**b**) the addition of the cover slip; (**c**) the fate of the worm in the worm chip, whereby the worm is surrounded by micro-columns. Reprinted with permission from the Royal Society of Chemistry.

Wang et al. developed an array of micro-columns-based microfluidic system to study a worm’s response to a crowded environment (Figure 8) [50]. The array of columns in the microfluidic chip provides sufficient mechanosensation to the worm to prompt a crowding stress response. The distance between the array of micro-columns can be adjusted, to create an appropriately crowded environment so as to induce a stress response at the cellular level, such as translocation of the fluorescently tagged protein, DAF-16::GFP, into the nuclear subcellular location, which researchers can then analyze by imaging the live worm at different time points. Crowding induces stress, in turn, affects the physiological behavior.

Another interesting device developed by Chuang et al. exploits the electrotaxis behavior of worms to induce a physically active environment, producing a “worm treadmill” effect. The physical activity produced in this manner is equivalent to exercise and research shows that this effect can protect the worm from age-related cellular degeneration. In this worm chip, where the worms are subjected to an electric field, the worms are attracted towards the cathode via electrotaxis in the microchannels. The polarity of the electric field can be switched between the electrodes so that the worms are attracted to the new cathode in turn, thereby producing the treadmill effect [21].

The combination of microfluidic devices and optogenetics had taken our understanding of the functional relationship between proteins and worm behavior to a whole new level. Several *C. elegans* mutant worms exhibit uncoordinated behavior and researchers have mapped the gene responsible for such behavior. However, while Hwang et al. examined the clear relationship between several muscle proteins and their collaborative role in behavior through the analysis of muscle kinetics, contraction and relaxation by subjecting an ontogenetically manipulable worm in microfluidic channels, other methods of obtaining such data do not exist [53].

### 4.4. Drug Screening and Toxicological Studies

*C. elegans* is a model organism with a genome ~65% similar to human disease genes [54,55]. For this reason, researchers use it for drug screening and toxicity examination of various chemical compounds. Varieties of worm chips are developed for real-time drug identification, screening and for testing toxicity based on worm behavior [56,57,58,59], electrophysiological signals [60], aging indications [61], antimicrobial or metabolic activity [62] and physical toxicity [63]. Thus, researchers use microfluidic systems not only on *C. elegans*, but also on other worm parasites for therapeutic agent development [56,58].

**Figure 9:**
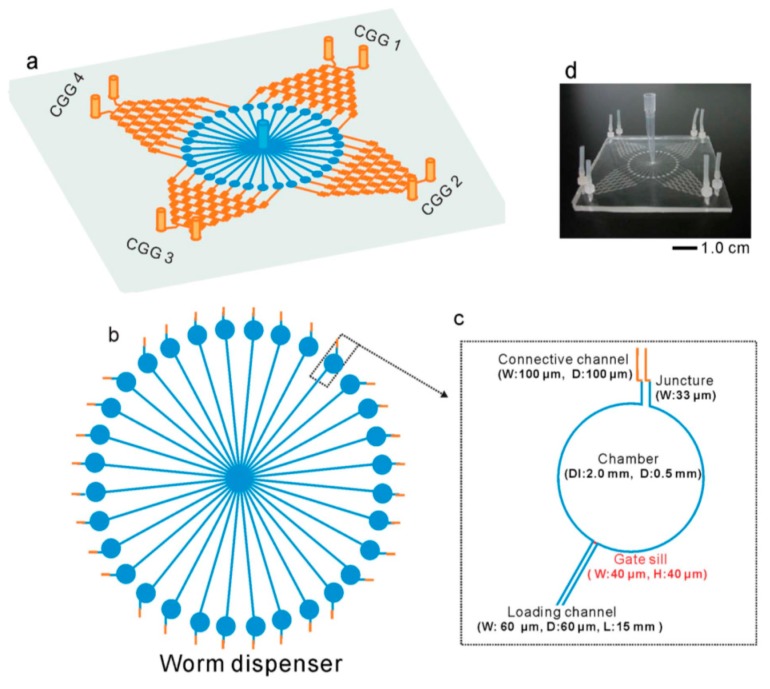
(**a**–**c**) Pictorial representations of the worm chip designed by Yang et al. [62] for drug screening. CGG 1 to 4 represent the concentration gradient generators, which generate different concentrations of drugs and whose channels connect to a chamber that radiates from a central reservoir, along with its dimensions; (**d**) the fabricated microfluidic worm chip. Reprinted with permission from the Royal Society of Chemistry.

Yang et al. developed a worm chip for screening and evaluating in vivo antimicrobial activity. The authors fabricated two layers of radial worm chips, radiating from a central reservoir to 32 chambers, which in turn were connected to drug delivery inlets from four concentration gradient generators (CGG). This worm chip can simultaneously screen 32 concentration gradients with four types of drugs (Figure 9). One can load the worms from the central reservoir, culture them in the chamber and analyze them simultaneously [62].

### 4.5. Microsurgery

Understanding the factors that play vital roles in neuronal regeneration has gained importance in the search of cures for several forms of neurodegenerative diseases and neuronal injuries. Researchers have developed *C. elegans* as one of the model organisms for studying the neuronal regeneration process, using femtosecond laser pulses to create precise ablation of the neurons [64]. Researchers are constantly developing and automating various microfluidic devices for worm handling so as to avoid the use of any anesthesia for neuronal axotomy [65,66]. One can also use some of the abovementioned worm chips for the immobilization of worms to immobilize worms for laser axotomy (refer to the “immobilization and imaging” section, Lee et al. [41]). Here, we can briefly outline a new automated microfluidic chip for laser microsurgery in worms. Gokce et al. designed a two-layer microfluidic system, in which the bottom layer, called the flow layer, transports the worm to different areas of the devices for immobilization, laser axotomy and removal, etc. The top layer is called the control layer and its primary function is to immobilize the worm through controlled valves upon pressurization. One can mount the chip on the microscope stage to perform laser severing using a femtosecond laser with a wavelength of 802 nm and 1 KHz pulsing frequency. The system connects to a pressurized external fluid chamber in order to control the flow rate into the microfluidic chip. The system is fully automated and one can control it via custom-written code, run by LabVIEW (National Instruments, Austin, TX, USA) [66].

### 4.6. Worm/C. elegans Sorting

Well-established genetic analysis has proven *C. elegans* to be a powerful genetic tool. Screening of new genes using both forward and reverse genetic screening is now a popular technique for understanding molecular pathways and protein functions in several genetic laboratories. Screening for a particular phenotype among several hundreds or thousands of worms is very laborious and time-consuming, but microfluidic systems have the power to greatly reduce the screening time and enable quick recovery/sorting of the observed genetic variant. The operation of worm sorting chips is based on various characteristic phenotypes of the worms, such as the visible microscopic phenotype of genetic variants, or the electrotaxis, behavioral phenotype, size and motility behavior of the worm, etc. Besides sorting worms after genetic screening [67,68], researchers use worm sorting chips to sort worms based on age [69], size [70,71], sex, motility [72], electrophysiological characteristics [73,74], etc. Researchers can make use of these different types of sorting in various metabolic, molecular biology and population studies.

Aubry et al. described a device (see the “Immobilization and Imaging” section) that can also be used for sorting worms that possess microscopic visible phenotypic variations after screening (Figure 2 and Figure 3) [38]. Aubry et al. characterized this method of sorting as “active” sorting of worms, whereby a researcher or computerized system actively identifies the worm, based on a fluorescent signal, that they must separate from the population of worms.

**Figure 10:**
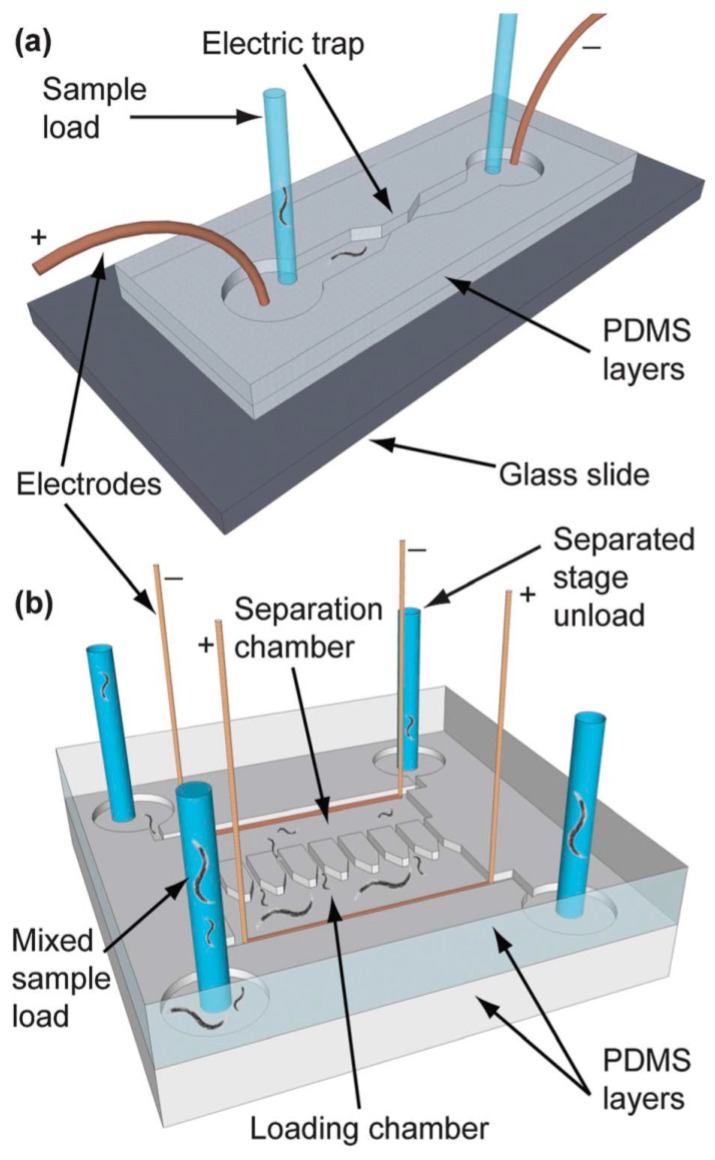
(**a**) The single electrotaxis worm sorter chip developed by Rezai et al. [73] with descriptions of its different parts; (**b**) a continuous electrotaxis worm sorter using the electric trap principle. Reprinted with permission from the Royal Society of Chemistry.

Rezai et al. described a single-layer microfluidic chip for worm sorting based on the electrotaxis behavior of the worms (Figure 10). This device can efficiently sort wild type worms at different developmental stages and screen for genetic mutant variants, which display defective neuronal and muscular activity as compared to wild type populations. Thus, the device can age synchronize the worms while screening for genetic mutation with defective neuronal and muscle activity [73]. The authors describe a passive method for sorting the worms. Unlike active sorting, the Rezai et al [73] device involves the separation of worms solely based on the differential attraction of worms towards an electrical signal, rendering this approach a passive sorting method. Yet another device designed by Casadevall I Solva et al [69]. works passively for age- and size-based synchronization of worms, and can achieve a rate of 200–1200 worms per minute with differential efficiency [69]. This device consists of pillar arrays, pools, smart filters and mazes in the flow chamber. As the worms pass through these obstacles they are segregated such that, for example, only larval worms are sorted into one particular channel, while adults are sorted into another.

**Figure 11:**
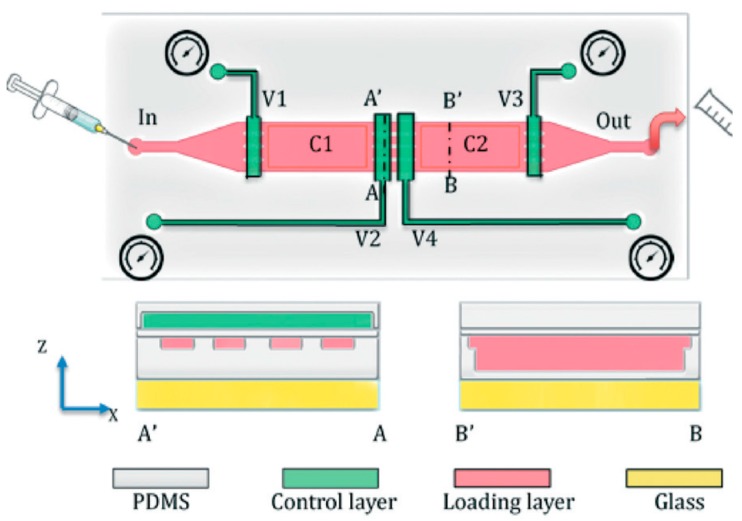
Schematic representation of the microfluidic device developed by Dong et al. [71] illustrating the two layers in the chip (control layer and flow layer) and their different parts. Reprinted with permission from the Royal Society of Chemistry.

Although there are different types of microfluidic chips available for size-dependent sorting of worm populations, each device varies in its structural design, design complexity, efficient worm handling and rate of sorting. Dong et al. designed a two-layer microfluidic chip in which an external pressure-based deformation property on a PDMS layer sorts the worms, based on the size of the space between the two chambers (Figure 11) [71]. Changes in the pressure of the control layer cause appropriate changes in the size of the channel in the PDMS flow layer, thereby allowing only those worms of a particular size to pass between the channel-separated chambers. It is necessary to standardize the precise optimization of pressure for particular worm size. The size-dependent sorting efficiency of this chip is 3.5 worms per second.

## 5. Challenges and Future Prospects

The development of microfluidic devices is a rapidly growing field and researchers have designed several varieties of such devices. Yet, to date, there is very limited commercial availability of microfluidic devices. Investors and sponsors should be involved in the development of these systems alongside researchers, so that they can bring forth strategies for making these devices commercially available. Recently, there has been a rapid increase in the number of research publications employing *C. elegans* as model organisms, which indicates that there is a growing number of new research groups around the world focusing on *C. elegans*. Therefore, making microfluidic devices commercially available would facilitate the research output, not only of worm biology, but also other fields.

In the future, microfluidic devices based on *C. elegans* that are integrated with the development of HTP microfluidic platforms will not only increase the number of samples analyzed but also reduce the amount of time such experiments require, resulting in a better understanding of these scientific subjects. The genetics of *C. elegans* have been well established and thus researchers widely employ this model organism as a genetic screening tool to identify novel genes involved in various cellular pathways. To put it simply, a HTP microfluidic platform for the efficient screening of genetic variants would greatly enhance the advancement of *C. elegans* research in the future. Besides enhancing our understanding of basic biology, researchers can also use *C. elegans* as a drug screening model or a toxicology model so as to develop therapeutically active ingredients or to identify toxic chemicals, respectively. As the development of HTP devices gains popularity for rapid drug screening purposes, there is ample opportunity for developing different types of HTP microfluidic devices that can increase the rate of therapeutic and toxicological screening, resulting in quick identification of potential therapeutic agents for common diseases and toxic substances.

## 6. Conclusions

Microfluidic devices have facilitated great advancements in scientific understanding. Not only useful in *C. elegans* research, these devices have also served as useful research tools for the study and decoding of several research models and model organisms, such as cultured cells, *Drosophila*, zebrafish, etc. [7,8,9,10]. Some microfluidic devices are even designed in such a way that they can be used for multiple model organisms [7,8,9,10]. It can be argued that microfluidic systems have potentially enhanced the efficiency of worm manipulation and handling in a user-friendly way for research laboratories. In addition, researchers can apply the basic findings in *C. elegans* biology using microfluidic devices to higher model organisms and, further, to medical therapeutic research.

As the scientific community increasingly demands ultra-HTP screening, data collection and analysis in pursuit of better understanding of biological phenomena, microfluidic/nanofluidic devices hold the “key” to facilitating such scientific advancement, for the benefit of future generations of scientists and society as a whole. This review article has emphasized the role of microfluidic systems in worm research and demonstrated how different designs of microfluidic devices can facilitate different study purposes, such as immobilization, imaging, behavioral analysis, screening, sorting, etc., using examples of recently designed fluidic chips for worm handling. Additionally, this article has outlined how findings in a model organism can be used for identification of therapeutic targets, thereby serving the human community.
